# Gut Bacterial and Fungal Communities of the Wild and Laboratory-Reared *Thitarodes* Larvae, Host of the Chinese Medicinal Fungus *Ophiocordyceps sinensis* on Tibetan Plateau

**DOI:** 10.3390/insects12040327

**Published:** 2021-04-07

**Authors:** Guiqing Liu, Xuehong Zheng, Hailin Long, Zhongchen Rao, Li Cao, Richou Han

**Affiliations:** Guangdong Key Laboratory of Animal Conservation and Resource Utilization, Guangdong Public Laboratory of Wild Animal Conservation and Utilization, Institute of Zoology, Guangdong Academy of Sciences, Guangzhou 510260, China; liugq@giz.gd.cn (G.L.); zxh1234560716@163.com (X.Z.); hlin_long@163.com (H.L.); xipredus@163.com (Z.R.); caol@giz.gd.cn (L.C.)

**Keywords:** ghost moth, *Thitarodes*, *Ophiocordyceps sinensis*, microbiota, high-altitude, artificial rearing

## Abstract

**Simple Summary:**

The ghost moth, *Thitarodes* sp., is an obligate host of the most precious fungus *Ophiocordyceps sinensis* on Tibetan plateau. Artificial rearing of the ghost moth at low-altitude laboratory by mimicking the environment of the wild habitat for the cultivation of the Chinese cordyceps has been realized. However, the high mortality of ghost moth larvae by pathogens, low and slow infection, and mummification rate by *O. sinensis* still constrain the efficient cultivation of the Chinese cordyceps. Both larval gut microbiota and their exploitation in the *Thitarodes* artificial rearing system have attracted a renewed interest. In the present study, the gut bacterial and fungal communities of the wild and laboratory-reared populations were characterized using both culture-dependent and -independent approaches. The discovery of apparent microbial community shifts between the wild and laboratory-reared ghost moth larvae, many opportunistic pathogenic bacteria and fungi in the gut of the laboratory-reared ghost moth larvae, and the dominant bacteria enriched in the wild ghost moth provide interesting cues for selecting beneficial probiotic bacteria to improve the effectiveness of *Thitarodes* rearing system and the cultivation of the Chinese cordyceps.

**Abstract:**

By employing a culture-dependent and -independent 16S rRNA and ITS gene high-throughput sequencing analyses, comprehensive information was obtained on the gut bacterial and fungal communities in the ghost moth larvae of three different geographic locations from high-altitude on Tibet plateau and from low-altitude laboratory. Twenty-six culturable bacterial species belonging to 21 genera and 14 fungal species belonging to 12 genera were identified from six populations by culture-dependent method. *Carnobacterium maltaromaticum* was the most abundant bacterial species from both the wild and laboratory-reared larvae. The most abundant OTUs in the wild ghost moth populations were Carnobacteriaceae, Enterobacteriaceae for bacteria, and Ascomycota and Basidiomycota for fungi. Larval microbial communities of the wild ghost moth from different geographic locations were not significantly different from each other but significant difference in larval microbial community was detected between the wild and laboratory-reared ghost moth. The larval gut of the wild ghost moth was dominated by the culturable *Carnobacterium*. However, that of the laboratory-reared ghost moth exhibited significantly abundant *Wolbachia*, *Rhizobium*, *Serratia*, *Pseudomonas,* and *Flavobacterium*. Furthermore, the larval gut of the wild ghost moth had a significantly higher abundance of *Ophiocordyceps* but lower abundance of *Candida* and *Aspergillus* than that of the laboratory-reared ghost moth.

## 1. Introduction

*Thitarodes/Hepialus* ghost moths (Lepidoptera: Hepialidae) are obligate hosts of the medicinal fungus *Ophiocordyceps sinensis,* and the fungus-insect parasitic complex named Chinese cordyceps has been one of the most valued health foods and traditional Asian medicines since the 15th century ([1,2,3]. Ghost moths belonging to the primitive lepidopteran lineage [4] are endemic to the Tibetan plateau in alpine and subalpine regions at altitudes from 3000–5200 m with a specific high-altitude environmental condition characterized by hypoxia, low air pressure, low temperature, and high ultraviolet radiation intensity [5,6,7]. The ghost moth larvae mainly inhabit subterranean tunnels built by themselves around the plant roots for more than 3 years [8,9,10]. The larvae feed mainly on plant roots underground, maintain the feeding activity at 0 °C or a few degrees above zero, and encounter various pathogens such as fungi, bacteria, nematodes, predatory mites, and some other small insects [11,12,13].

The underlying mechanism of the adaptation to the high-altitude environment of the ghost moth, and their coexistence with the fungus *O. sinensis* has attracted much attention in recent years. Transcriptome analyses using high throughput sequencing have provided in-depth insights into the mechanism of environmental adaptation. In response to cold, metabolic rate and respiratory quotient decrease [14]. Differentially expressed genes associated with altitude were annotated in the process of lipid metabolism, carbohydrate metabolism, and respiration [15]. The ghost moth *T. armoricanus* might adopt a strategy to adapt to hypoxia by suppressing hypoxanthine catabolism, TCA, and oxidative phosphorylation pathways [16]. In response to *O. sinensis* infection, only a proportion of infected larvae could turn into stiff worms and become Chinese cordyceps even though the larvae were sampled from the same site or reared at the same conditions [17,18]. It appeared that the immune system and gut microbiota are involved in the pathogenicity of *O. sinensis* fungus [19,20,21].

Insect gut is the house of diverse microbiomes, which are evolving to play important roles in insect physiology and ecology, supporting growth, development, and survival of their hosts [22,23,24]. In general, gut microbiota can contribute to the nutrition provision for host [25], host immunity [26], host behavior [27], and mediate detoxification of plant secondary compounds [28,29] and insecticides [30]. Conversely, multiple factors such as host, environment, and diet can shape the structure of gut microbial communities [31,32,33,34,35]. Nevertheless, all these studies are based on the gut microbiota of low-altitude insect hosts.

So far, artificial rearing of the ghost moth at low-altitude laboratory by mimicking environment conditions of the wild habitat for the cultivation of the Chinese cordyceps at commercial scale has been successfully established [3,36]. However, the high mortality of ghost moth larvae by pathogens, low and slow infection, and mummification rate by *O. sinensis* still constrain the efficient production of the Chinese cordyceps [2,37,38]. Given the important role of the gut microbes, researchers attempt to identify microbes from the gut of the ghost moth larvae, hoping to find beneficial microbes for the artificial rearing ghost moth larvae and promoting the infection and mummification rate by *O. sinensis*. However, only 8 bacterial and 3 fungal genera were isolated from the wild *T. gonggaensis* larvae [39,40] and 11 bacterial and 3 fungal genera were isolated from the laboratory-reared *T. xiaojinensis* [21] by the culture-dependent method. Furthermore, only *Carnobacterium* and *Pseudomonas* were common in the wild *T. gonggaensis* larvae and the laboratory-reared *T. xiaojinensis*. Therefore, it is necessary to comprehensively compare the gut microbiota between the wild and laboratory-reared ghost moth.

To explore the involvement of the gut microbiota in host physiology and pathogenicity, the larval gut bacterial and fungal communities of the wild ghost moth collected at alpine meadow on Tibet plateau and laboratory-reared ghost moth among three different geographic locations were characterized using both culture-dependent and culture-independent approaches.

## 2. Materials and Methods

### 2.1. Ghost Moth Collection, Identification and Gut Preparation

The wild ghost moth 5th instar larvae (average fresh weight = 0.49 ± 0.08 g) were sampled from the known distribution areas of the Chinese cordyceps. Samples were collected from soil beneath alpine meadow from three different geographic locations during the harvesting seasons (May–July 2019) at altitudes 3500 m above sea level on the Tibetan plateau in Sichuan Province, China (See Figure S1 for details). The larvae from the above three different locations were named W.SD, W.GG and W.XJ populations, respectively according to their geographic location (Figure S1). The alive larvae were collected and placed individually in the vial filled with moss and sent to the laboratory in foam boxes with ice packs to maintain the low temperature.

The laboratory-reared ghost moth larvae were derived from the pupae collected from the above three geographic locations and reared at 9~13 °C in Guangzhou, China according to the described methods [21,41]. The hatched larvae were offered the roots of *Potentilla anserine* as food to obtain 5th instar larvae (average fresh weight = 0.57 ± 0.07 g) for gut microbial isolation. The laboratory-reared larvae from the above three different locations were named as A.SD, A.GG, and A.XJ populations, correspondingly.

To identify the ghost moths, the genomic DNA of the wild and laboratory-reared ghost moths were extracted using TaKaRa MiniBEST Universal Genomic DNA Extraction Kit (Takara Bio Inc, Dalian, China) and used as a template to amplify the mitochondrial cytochrome b gene (cytb) with the primers CB1 (TATGTACTACCATGAGGACAAATATC) and CB2 (ATTACACCTCCTAATTTATTAGGAAT) [42]. Then, the phylogenetic tree of the ghost moths from three different locations was constructed using neighbor-joining method in the MEGA 5.1 with a bootstrap value of 1000.

All the larvae were dissected within 3 days after collection. The larvae were surface-sterilized with 75% ethanol for 60 s and rinsed three times with sterilized deionized water. The dissection was performed on ice under sterile conditions. Twenty larval guts were pooled as one sample and three samples were established for each population. Isolated gut contents were immediately placed in precooled Eppendorf tubes and homogenized with 1 mL sterile 1×phosphate buffered saline (PBS, pH 7.2). All the samples were divided into two parts, one for culture-dependent method and the other for amplicon sequencing. Samples used for amplicon sequencing were flash-frozen with liquid nitrogen and homogenized with a pestle to extract DNA.

### 2.2. Culture-Dependent Microbial Communities

For cultivation experiment, the gut content suspension was diluted into 10^−4^, 10^−5^, and 10^−6^ dilution series with sterile 1 × PBS, and an aliquot of 100 µL of the suspension was spread on plates of LB (Lucia-Bertani), TSB (Trypticase Soy Broth), GSA (Gause’s synthetic agar; HKM, Guangzhou, China), HIA (Heart infusion agar; BD, Baltimore, Maryland, USA), and PPDA [18,43] to isolate the bacteria and fungi. Twelve plates were set up for each treatment. All the plates were sealed with parafilm (BEMIS, Ninah, Wisconsin, USA) and then cultured in the dark condition at 13 °C and 25 °C, respectively.

The growing colonies of bacteria and fungi on the plates were screened on the basis of colony appearance and further purified by quadrant streaking on LB or PPDA plates, respectively. To identify the microbe isolates, the prokaryotic V4 region of 16S rRNA was amplified with primers 27F (5′-AGAGTTTGATCCTGGCTCAG-3′)/1492R (5′-TACGGYTACCTTGTTACGACTT-3′) for bacteria and the ITS2 region of ITS gene was amplified with ITS5 (5′-GGAAGTAAAAGTCGTAACAAGG-3′)/ITS4 (5′-TCCTCCGCTTATTGATATGC-3′) for fungi, respectively. The PCR products were separated by electrophoresis and the bands were cut from the gels for purification, and then sequenced in Sangon Biotech (Shanghai) Co., Ltd. (Guangzhou, China). Sequence analysis was performed using nucleotide blast http://www.ncbi.nlm.nih.gov/ (accessed on 1 November 2019). The representative sequences of each bacterial and fungal species were submitted to GenBank https://www.ncbi.nlm.nih.gov/genbank/ (accessed on 2 February 2020) under accession numbers MW555179-MW555204 and MW555208-MW555221, respectively.

### 2.3. Culture-Independent Microbial Communities

Total DNA was extracted from each pool using the QIAamp DNA Stool Mini Kit (Qiagen, Hilden, Germany) according to the manufacturer’s instructions. 16S rRNA and ITS gene amplicons of the gut samples were produced and sequenced using an Illumina Nova 6000 platform (Guangdong Magigene Biotechnology Co., Ltd. Guangzhou, China). Briefly, the V5–V6 region of the 16S rRNA gene was amplified using primers 799F (5′-AACMGGATTAGATACCCKG-3′)/1193R (5′-ACGTCATCCCCACCTTCC-3′) with 12bp barcode. The ITS1 region of the fungal ITS gene was amplified using the primers BD-ITS1F (5′-GGAAGTAAAAGTCGTAACAAGG-3′)/ITS2-2043R (5′-GCTGCGTTCTTCATCGATGC-3′) with 12 bp barcode.

The sequencing data were qualified using Fastp (version 0.14.1). To obtain the paired-end clean reads, the primers were removed by using cutadapt software (https://github.com/marcelm/cutadapt/ (accessed on 2 March 2020). Paired-end clean reads were merged using usearch -fastq_mergepairs (V10http://www.drive5.com/usearch/ (accessed on 2 March 2020). Raw tags were merged when at least 16 bp overlap the read generated from the opposite end of the same DNA fragment, with the maximum mismatch allowed in overlap region being 5 bp. Clean Tags were generated after removal of barcodes and primers. The resulting high-quality reads were clustered into operational taxonomic units (OTU) by usearch-sintax (set the confidence threshold to default to ≥0.8) using SILVA (V119, http://www.arb-silva.de (accessed on 2 March 2020) for bacteria and UNITE 252 V7.0, http://unite.ut.ee/index.php (accessed on 12 March 2020) for fungi. After removal of the OTU and its Tags, which were annotated as chloroplasts or mitochondria (16S amplicons) and not annotated to the kingdom level, the final OTU taxonomy synthesis information table was obtained.

For each sample, alpha diversity was applied to estimate complexity of species for a sample by calculating bacterial and fungal alpha diversity indices, including richness, chao 1, and Simpon’s index. All these indices were calculated with usearch-alpha_div (V10, http://www.drive5.com/usearch/ accessed on 2 March 2020) and calculated rarefaction curve and rank abundance separately. The differences between groups were analyzed by alpha diversity indices using Student’s t-test for two groups and Kruskal Wallis for more than two groups (*p* = 0.05) with R software. Heat maps at the phylum, family, and genus levels among the six groups were generated with R using *ampvis2* package [44].

Beta diversity was measured by Principal Coordinate Analysis (PCoA) based on Bray-Curtis distance matrixes and was displayed by vegan package in R Software. In addition, unweighted pair-group method with arithmetic means (UPGMA) clustering analysis was also performed to interpret the distance matrix using average linkage. To analyze the difference of community structure between groups, permutational multivariate analysis of variance (Permanova) statistical analyses were conducted based on Bray-Curtis distance matrixes with 999 permutations using two non-parametric analyses including analysis of similarity (Anosim) and non-parametric multivariate analysis of variance (Adonis) of vegan and pegas package in R software.

To identify microbes accounting for the effects of geographic locations and altered habitat conditions, the linear discriminatory analysis (LDA) effect size (LEfSe) algorithm was used to compare the differential abundances of bacteria and fungi among groups at family and genus levels using LEfSe software [45]. Non-parametric factorial Kruskal Wallis sum rank test was used to identify taxa with significant difference in abundance among groups (*p* < 0.05). Then, Wilcoxon rank sum test was used to investigate biological consistency among subgroups. Finally, LDA was used to estimate the impact of each selected taxon. Only those taxa with more than four orders of magnitude (LDA score > 4) were considered in this study.

All obtained 16S and ITS amplicon data have been deposited in the Sequence Read Archive (SRA) under accession number PRJNA698401.

## 3. Results

### 3.1. Molecular Identification of the Ghost Moth Populations

The information on six groups (W.SD, W.GG, W.XJ, A.SD, A.GG, and A.XJ) including sampling altitude (masl, meters above sea level), environmental temperature, major diet, and location were presented in Table 1. Larvae derived from Xiaojin County (W.XJ and A.XJ) were identified as *Thitarodes xiaojinensis* by the mitochondrial cytochrome b sequence. The phylogenetic tree of the ghost moths from the above three locations constructed by the mitochondrial cytochrome b gene showed that population W.SD was closely related to population W.GG (Figure S2).

### 3.2. Culture-Dependent Communities

Overall, 26 bacterial species belonging to 21 genera and 14 fungal species belonging to 12 genera were identified from the gut of the ghost moth larvae from the six populations. At the phylum level, 11, 5, 9, and 1 species of 26 bacterial species were assigned to Proteobacteria, Firmicutes, Actinobacteria, and Bacteroidetes, respectively. For fungi, 9, 3, and 2 species were assigned to Ascomycota, Basidiomycota, and Mucoromycota, respectively. Four culturable bacterial species including *Carnobacterium maltaromaticum*, *Rahnella aquatilis*, *Pseudomonas* sp., and *Streptomyces* sp. were considered as shared species, among which *C. maltaromaticum* was the most abundant bacterial species cultivated from both the wild and laboratory-reared samples (Table 2).

Compared with the laboratory-reared larvae, more colonies of *C. maltaromaticum* were detected from the wild ghost moth larvae. *Raoultella terrigena* was shared in the wild samples collected from the three different locations but not detected in the laboratory-reared populations. *Agromyces* sp. and *Pseudoclavibacter* sp. were only detected in the wild larvae from SD population, while *Oerskovia* sp. and *Pantoea* sp. were only detected from GG population and *Buttiauxella* sp. were only detected from XJ population. Similarly, more fungal species appeared from the wild larvae and no fungal species were shared in all six populations (Table 2).

The percentages of the microbe species obtained from the above five different media at two different temperatures are shown in Table S1. Generally, more bacterial and fungal species were isolated from the plates containing TSB and PPDA media at 23 °C.

### 3.3. Culture-Independent Communities

A total of 1,571,246 high-quality clean reads of bacteria (16S rRNA), and of 1,578,989 high-quality clean reads of fungi (ITS) were produced from the six populations with 3 replicates for each population (Table S2), indicating high quality sequencing. Each sample contained at least 60,000 effective sequences for 16S and ITS amplicons (Figure S3). The rarefaction curves for all samples showed that the sequence depths were reliable for both bacterial and fungal identification in each sample (Figure S4).

#### 3.3.1. General Pattern of the Gut Microbiota of the Wild Ghost Moth Populations

**Figure 1:**
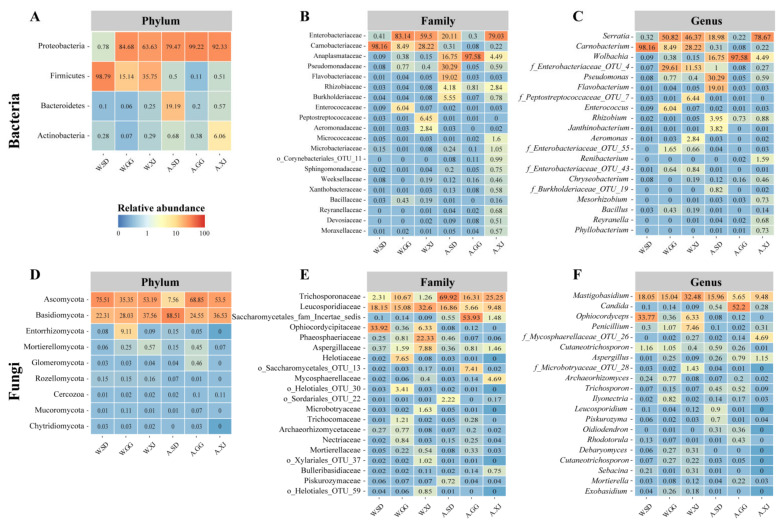
The bacterial and fungal communities of the wild and laboratory-reared ghost moth larvae. Heatmaps of the bacterial (**A**) and fungal (**B**) top phyla, bacterial (**C**) and fungal (**D**) top 20 family, and bacterial (**E**) and fungal (**F**) top 20 genera based on the relative abundance of OTUs in all samples. Relative abundance is the mean of replicate samples.

In the total dataset, most of the bacteria were identified as Firmicutes (98.79, 15.14 and 35.75% in the gut of the wild SD, GG and XJ, respectively), followed by Proteobacteria (0.78, 84.68 and 63.63% in the wild SD, GG and XJ) and to a lesser extent, Bacteroidetes and Actinobacteria (Figure 1A). At the family level, the most abundant bacterial taxa in the wild SD were Carnobacteriaceae. However, the wild GG and XJ were dominated by Enterobacteriaceae, followed by Carnobacteriaceae (Figure 1B). At the genus level, the wild SD bacterial community was dominated by *Carnobacterium*, while the wild GG was dominated by *Serratia*, *f-Enterobacteriaceae_OTU_4*, *Carnobacterium***,** *Enterococcus,* and *f-Enterobacteriaceae_OTU_55*. The most frequently occurring genera from the wild XJ were *Serratia*, *Carnobacterium*, *f-Enterobacteriaceae_OTU_4*, *f-Enterobacteriaceae_OTU_7*, and *Aeromonas* (Figure 1C).

For fungi, the wild SD and XJ populations were dominated by Ascomycota (Comprising 75.51 and 53.19%, respectively), followed by Basidiomycota (Comprising 22.31 and 37.56%, respectively), and the wild GG population was dominated by Ascomycota (35.35%), Basidiomycota (28.03%), and Entorrhizomycota (9.11%) (Figure 1D). At the family level, the most abundant fungal taxa in the three wild populations were different, Ophiocordycipitaceae, Leucosporidiaceae, and Trichosporonaceae in the wild SD; Leucosporidiaceae, Trichosporonaceae, Helotiaceae, and Aspergillaceae in the wild GG; and Leucosporidiaceae, Phaeosphaeriaceae, Aspergillaceae, Ophiocordycipitaceae, Microbotryaceae, and Trichosporonaceae in the Wild XJ (Figure 1E). The most frequently occurring genera of the wild SD population were *Ophiocordyceps*, *Mastigobasidium,* and *Cutaneotrichosporon*. The most frequently occurring genera of the wild GG population were *Mastigobasidium*, *Penicillium*, *Cutaneotrichosporon*, *Ilyonectria,* and *Archaeorhizomyces*. The most frequently occurring genera of the wild XJ population were *Mastigobasidium*, *Penicillium*, and *Ophiocordyceps* (Figure 1F).

**Figure 2:**
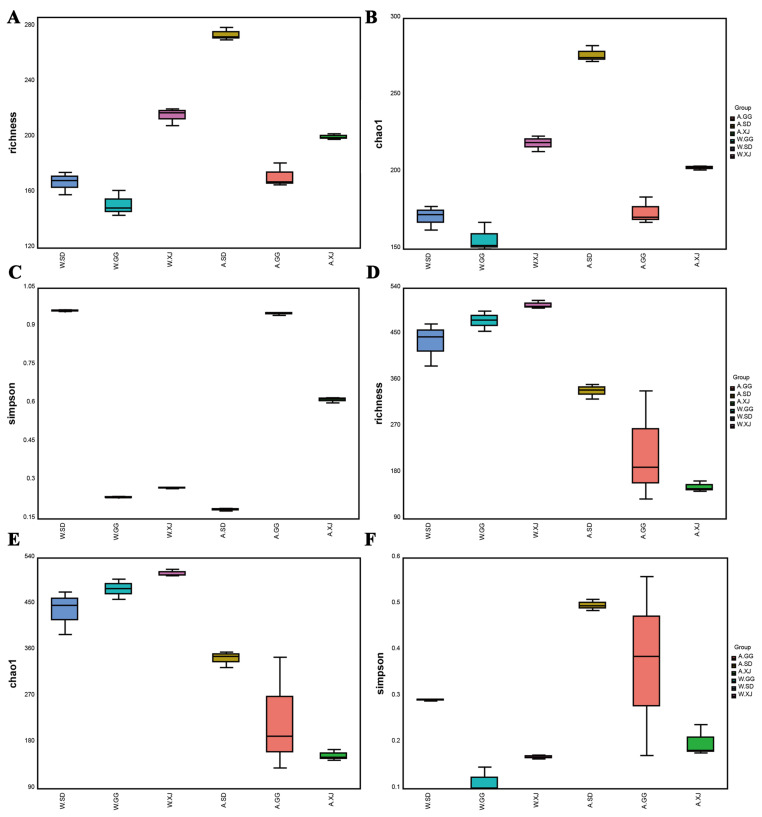
The alpha diversity of the gut microbial composition. Richness, Chao1, and Simpson indices of bacteria (**A**–**C**) and fungi (**D**–**F**). Richness means the number of OTUs determined with 16S rRNA gene (Bacteria) or ITS gene (Fungi) based on DNA extracted from populations; Chao 1 means the total number of OTUs estimated by infinite sampling, and a higher number indicates a higher richness [48]; Simpson’s index measures community evenness, and index increases as diversity decreases [49].

**Figure 3:**
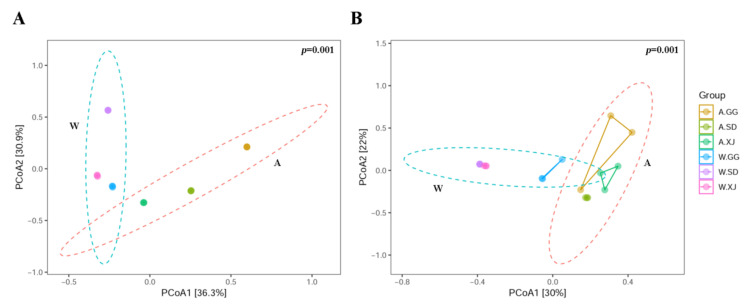
PCoA visualization using the Bray-Curtis dissimilarity measurement separating samples. PCoA analysis based on Bray-Curtis distance matrix for bacterial (**A**) and fungal (**B**) communities of all samples.

The Richness and Chao1 indices of the gut bacteria in the wild XJ were significantly higher than those in the wild SD and GG. The Simpson indices of the gut bacteria and fungi in the wild SD were significantly higher than those in the wild GG and the wild XJ, which indicated that the bacterial and fungal diversities of the wild SD were significantly lower than those of the wild GG and the wild XJ (Figure 2 and Table S3). However, the ANOSIM and Adonis analyses revealed no significant microbial community difference in the wild ghost moth collected from different geographic locations (Figure 3 and Table S4).

#### 3.3.2. Comparison of the Gut Microbial Community Diversity between Insect Populations

A comparison of alpha diversity indices between ghost moth populations was presented in Figure 2 and the significance was detected by turkey method. For bacteria, there were significant differences among the laboratory-reared ghost moths from SD, GG, and XJ populations (A.SD versus A.GG, A.SD versus A.XJ, and A.GG versus A.XJ). The bacterial richness and diversity of the laboratory-reared ghost moth from SD population were significantly higher than those from GG and XJ populations (Figure 2A–C and Table S3). In addition, the laboratory-reared ghost moth was notably dissimilar with the wild ghost moth (A.SD versus W.SD, A.GG versus W.GG, and A.XJ versus W.XJ). Artificial rearing of the larvae in the laboratory significantly increased the bacterial richness and diversity in SD population, but significantly decreased the bacterial diversity in GG and XJ population. For fungi, there was a significant difference between the laboratory-reared ghost moths from SD and XJ populations (A.SD versus A.XJ), but no detectable significant differences were found between A.SD and A.GG or A.GG and A.XJ. Compared with the wild ghost moth, artificial rearing significantly decreased the fungal richness and diversity in SD and XJ populations (A.SD versus W.SD and A.XJ versus W.XJ). However, no detectable significant difference was found between the laboratory-reared and wild ghost moth from GG population (A.GG versus W.GG) (Figure 2D–F and Table S3).

**Figure 4:**
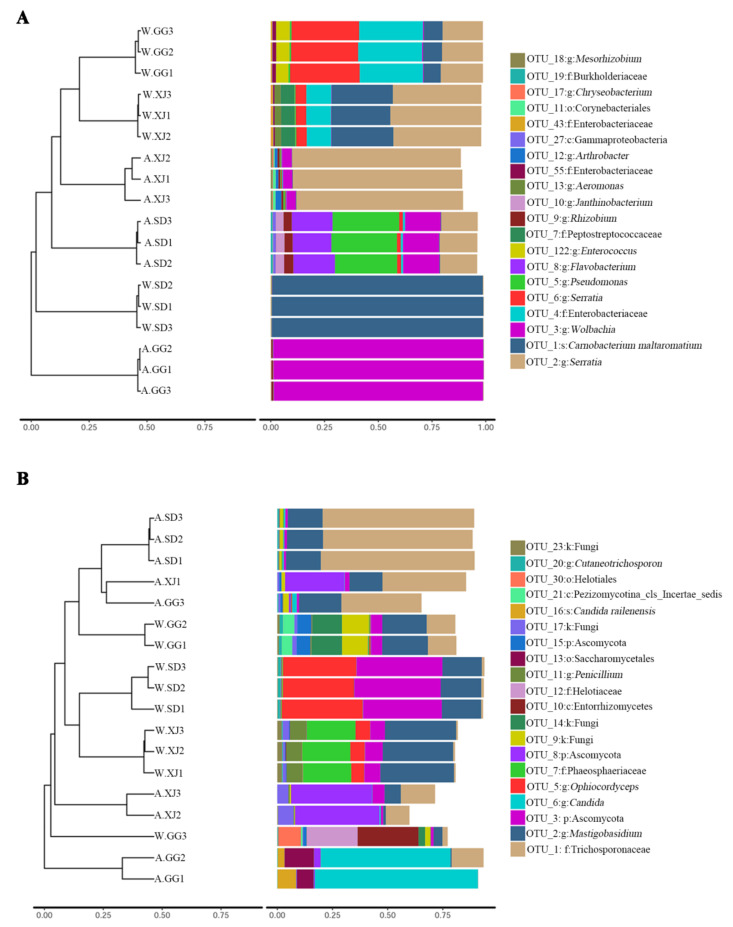
Clustering analysis of beta-diversity based on the Bray-Curtis distance matrix for bacterial (**A**) and fungal (**B**) communities of all samples by UPGMA method.

Similarities in the bacterial and fungal community compositions between populations were compared by PCoA based on the Bray-Curtcis index. The ANOSIM and Adonis analyses revealed significant microbial community difference between the wild and laboratory-reared samples (Figure 3 and Table S4). However, the wild and laboratory-reared populations from the same location tended to separate from each other for bacterial community diversity but the difference was not significant (Figure 3A and Table S4). For fungal community diversity, the wild SD and XJ populations were slightly separated from the laboratory-reared SD and XJ populations, respectively (Figure 3B and Table S4). Nevertheless, one wild GG sample (W.GG3) was separated from the other wild GG samples, and one laboratory-reared GG sample (A.GG3) was also separated from the other laboratory-reared GG samples. These differences were also revealed by UPMA clustering (Figure 4). The separation of sample W.GG3 from samples W.GG1 and W.GG2 was due to increased abundance of Helotiaceae and Saccharomycetales, while the separation of sample A.GG3 from samples A.GG1 and A.GG2 was due to decreased abundance of *Candia* and increased abundance of OTUs belonging to *Mastigobasidium* and Trichosporonaceae.

#### 3.3.3. Differential Microbes among Insect Populations

**Figure 5:**
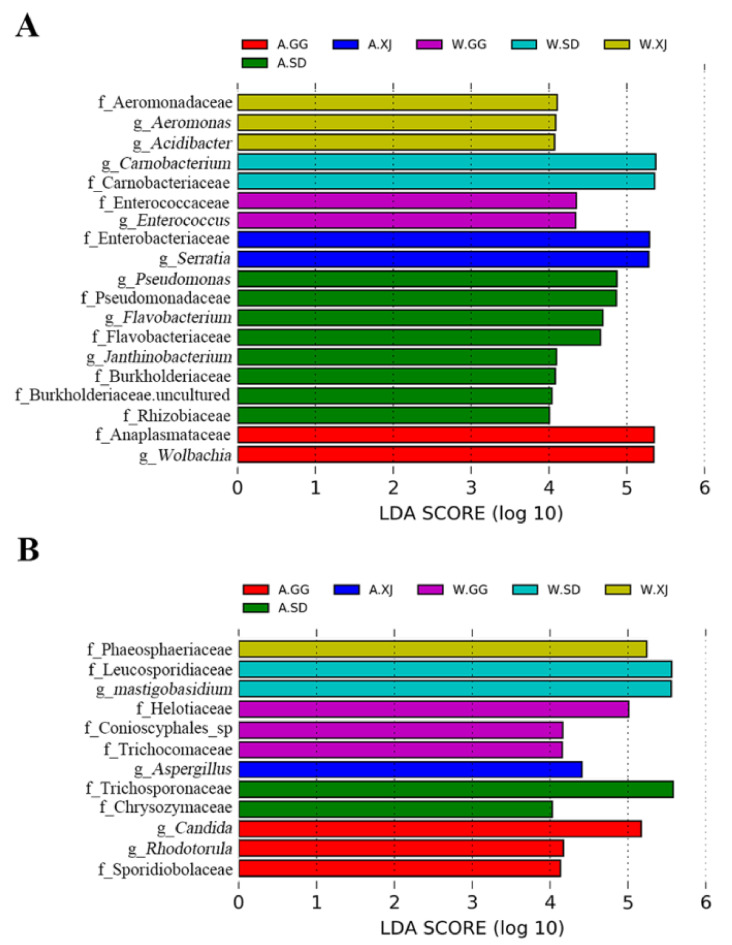
Differences in bacterial (**A**) and fungal (**B**) taxa among groups determined by linear discriminative analysis effect size (LEfSe). LDA scores could be interpreted as the degree of difference in relative abundance. Abbreviation: g_, genus and f_, family.

**Figure 6:**
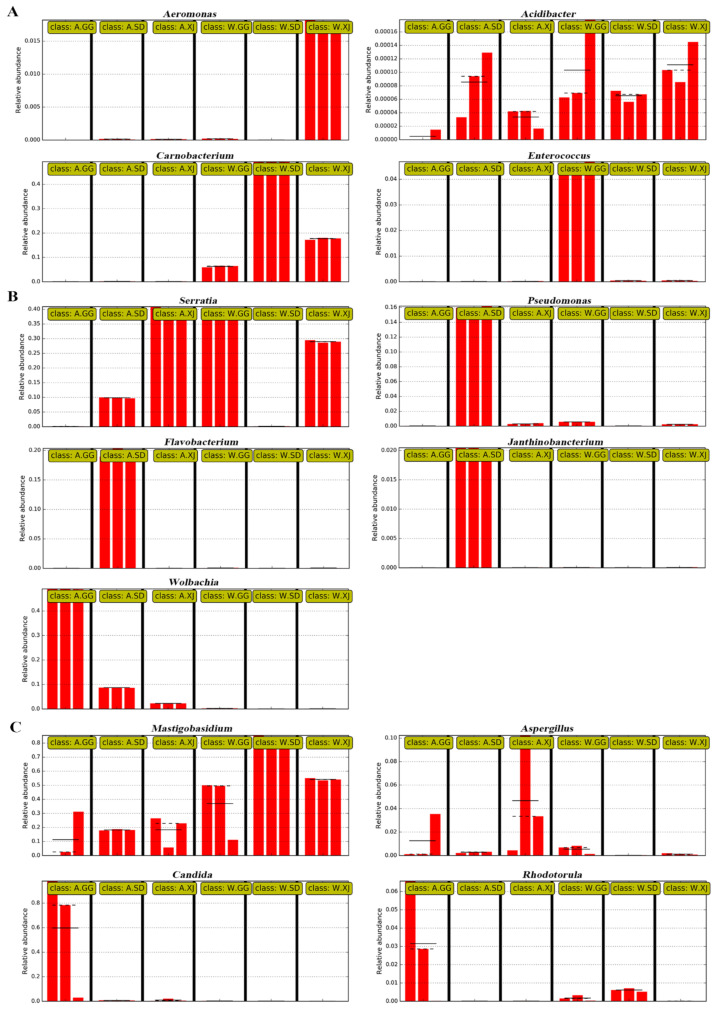
Relative abundances of differential microbes at the genus level selected by LEfSe analysis among populations. The straight lines indicate the mean. The dotted lines indicate the median. (**A**) Dominant bacteria in the gut of the wild ghost moth; (**B**) Dominant bacteria in the gut of the laboratory-reared ghost moth larvae; (**C**) Dominant fungal in the gut of the wild and laboratory-reared ghost moth larvae.

Based on the significant differences among the samples by using linear discriminant analysis (LDA), 9 genera and 10 families of bacteria and 4 genera and 8 families of fungi were significantly enriched in different populations (Figure 5). The relative abundance of each selected genus was presented in Figure 6.

When compared with the wild ghost moth, the gut bacteria of the laboratory-reared SD population showed increased abundance of genera *Pseudomonas*, *Flavobacterium*, and *Janthinobacterium* and families Pseudomonadaceae, Flavobacteriaceae, Burkholderiaceae, and Rhizobiaceae. The laboratory-reared GG population had a significant higher abundance of genus *Wolbachia* and family Anaplasmataceae, while the laboratory-reared XJ population showed increased abundance of genus *Serratia* and family Enterobacteriaceae (Figure 5A). For fungi, the laboratory-reared SD population showed increased abundance of families Trichosporonaceae and Chrysozymaceae, while the laboratory-reared GG population showed increased abundance of genera *Candida* and *Rhodotorula* and family Sporidiobolaceae. The laboratory-reared XJ population had a higher abundance of genus *Aspergillus* (Figure 5B).

**Figure 7:**
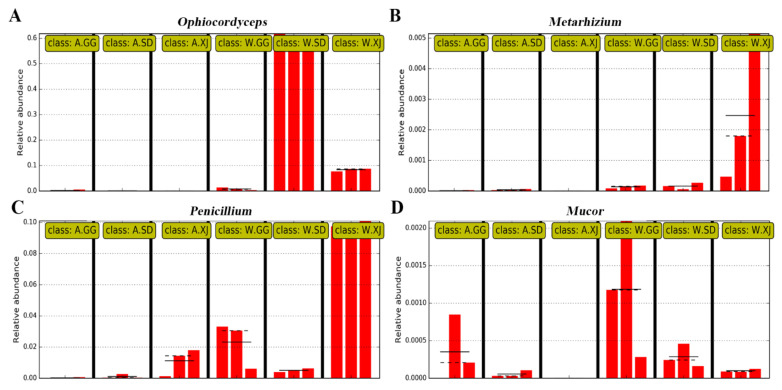
Relative abundance of the fungus isolated from the mummified laboratory-reared ghost moth larvae. (**A**) Relative abundance of *Ophiocordyceps*; (**B**) Relative abundance of *Metarhizium*; (**C**) Relative abundance of *Penicillium*; (**D**) Relative abundance of *Mucor*.

A lot of fungal pathogens could infect and kill the larvae, and the most fatal pathogen is *Isaria farinosa* (formerly *Paecilomyces farinosus*) based on the identification of isolates from the ghost moth cadaver killed by microbes during the artificial rearing of the ghost moth (Table S5). When checking the relative abundance of these fungal pathogens, *Ophiocordyceps*, which infected the ghost moth larvae to form the Chinese cordyceps, showed a higher relative abundance in the wild larvae, especially in the wild SD population than that in the laboratory-reared larvae (Figure 7A). Another entomopathogenic fungus *Beauveria bassiana* was present in the gut of both the wild and laboratory-reared larvae from SD population, but its abundance was very low. The entomopathogenic fungus *Metarhizium* showed increased abundance in the wild larvae from XJ population (Figure 7B). The wound pathogenic fungus *Mucor* and the saprophytic fungus *Penicillium* were also present in the gut of the wild and laboratory-reared ghost moths (Figure 7C,D). However, the most fatal entomopathogenic fungus *I. farinosa* was not detected in the gut of both the wild and laboratory-reared larvae, which was further demonstrated by PCR amplification using the specific primers [50] of *I. farinosa* (Figure S5).

## 4. Discussion

By employing a culture-dependent and -independent 16S rRNA and ITS gene high-throughput sequencing analyses, comprehensive information was obtained on the gut bacterial and fungal community in the ghost moth larvae of three different geographic locations from high-altitude on Tibet plateau and from low-altitude laboratory. The discovery of apparent microbial community shifts between the wild and laboratory-reared ghost moth larvae, many opportunistic pathogenic bacteria and fungi in the gut of the laboratory-reared larvae, and the enriched dominant bacteria in the wild ghost moth provide new insights to improve the effectiveness of the laboratory-reared *Thitarodes* hosts of *O. sinensis* for the cultivation of the Chinese cordyceps.

The culture-dependent method resulted in the isolation of 21 bacterial genera and 12 fungal genera from six ghost moth populations (Table 2). Compared with at most eight bacterial genera and three fungal genera isolated from the gut of the wild *T. gonggaensis* larvae [39,40,51], more bacteria and fungi were isolated in the present study, and seven bacterial genera *Acinetobacter*, *Aeromomonas*, *Bacillus*, *Carnobacterium*, *Pantoea*, *Pseudomonas,* and *Staphylococcus* were common. These isolated bacteria belonged to Proteobacteria, Firmicutes, Bacteroidetes, and Actinobacteria, and the fungi to Ascomycota, Basidiomycota, and Mucoromycota. Among the four shared bacteria isolated from six populations in this study, *C. maltaromaticum* were the most abundant culturable species both in the wild and laboratory-reared ghost moth larvae.

Taxonomic analysis revealed that the wild ghost moth gut bacterial community was dominated by Proteobacteria and Firmicutes (>99%), while the laboratory-reared ghost moth gut bacterial community was mainly composed of Proteobacteria with significantly decreased abundance in Firmicutes, but increased abundance in Bacteroidetes and Actinobacteria (Figure 1). These four phyla were also the most common found in other lepidopteran species [35,52,53,54]. In this study, the fungal community of the ghost moth populations primarily consisted of *Ascomycota* and *Basidiomycota*, which was similar to other lepidopteran insects [53].

The composition of the ghost moth gut microbiota was unique. Each wild ghost moth population appeared to harbor a unique complex of microbes in their guts. Different host and growing environment (different altitude, diet and temperature) might be the factors that resulted in the changes among the wild ghost moth populations. For each population, the changed gut microbial communities were observed in the laboratory-reared population and the changes of each population due to artificial rearing appeared to be consistent within each of the samples. However, different gut microbial communities were found in the three laboratory-reared populations even though they were exposed to the same rearing conditions.

The wild populations had high abundance of *Carnobacterium*, particularly, the abundance of *Carnobacterium* in the wild SD samples reached up to 98.16% (Figure 1). Because *Carnobacterium* was detected in all samples, it might be a commensal inhabitant of the ghost moth. This inference was also supported by the presence of *Carnobacterium* in *Thitarodes* sp. unfertilized eggs and larvae [21,39,55]. *Carnobacterium* is a genus of Lactic Acid Bacteria (LAB), and *C. maltaromaticum* is frequently isolated from natural environment and foods [56]. It is able to grow at low temperatures, anaerobically and with increased CO_2_ concentrations [57]. Thus, it is reasonable to explain its overwhelming dominance in the gut of the wild ghost moth larvae on Tibet plateau. However, it is unclear whether the changed environment resulted in the significant decrease in the abundance of *C. maltaromaticum* in the laboratory-reared ghost moth. In addition, previous studies have shown that *C. maltaromaticum* is a probiotic bacterium extensively used in fish, meat, and dairy products, which can inhibit pathogenic and spoilage microorganisms [57]. It can also out-compete the pathogen and modulate the autochthonous midgut microbiota of Atlantic cod *Gadus morhua* [58]. More colonies of *C. maltaromaticum* were also detected in the gut of *T. xiaojinensis* larvae uninfected with *O. sinensis* than those injected with *O. sinensis* [21]. Therefore, whether *C. maltaromaticum* could be used as a probiotic bacterium to improve the growth of the ghost moth larvae needs further study.

When compared with the wild ghost moth larvae, the gut bacteria of laboratory-reared ghost moth larvae showed a significant increase in the abundance of *Wolbachia* and *Rhizobium* (Figure 6). *Wolbachia* is commonly found in about 80% of lepidopteran species [59]. *Wolbachia* is an intracellular bacterium, which is usually found in reproduction tissues, but it can also be found in different host tissues such as ovary, testis, fat body, midgut, Malpighian tubule, and leg [60]. It is considered as a parasite that could manipulate reproduction in Lepidoptera [61] and has also been found to inhabit insect cells and restrict viral infection in *Drosophia melanogaster* [62] and *Aedes aegypti* [63]. However, factors that resulted in significantly increased abundance of *Wolbachia* in the laboratory-reared ghost moth, especially in GG population, and the function of *Wolbachia* probably involved in the artificial rearing process are still unknown. In addition, some gut-associated bacterial genera also detected the increased abundance in the laboratory-reared ghost moth, for instance, *Pseudomonas* and *Flavobacterium* in SD population, and *Serratia* in XJ population. These genera are opportunistic pathogens in animal and/or humans and have also been found to facilitate other pathogens to infect host insects [64,65]. Therefore, the increased abundance in the laboratory-reared ghost moth should be carefully considered.

The infection mechanism of *Thitarodes/Hepilus* larvae by *O. sinensis* fungus is not well establish [3,66]. In the present study, it is suggested that *O. sinensis* may enter *Thitarodes* host by feeding as shared and high abundance of *O. sinensis* was present in the gut of the wild ghost moth populations from different geographic locations. However, significantly decreased abundance of *O. sinensis* and increased abundance of opportunistic pathogens *Aspergillus* and *Candida* were found in the laboratory-reared ghost moth populations. Interestingly, the most common pathogen *I. farinosa* was not detected in the gut of both the wild and laboratory-reared ghost moth. It seems that this fungus is an opportunistic pathogen for ghost moth larvae, as reported by Wu et al. [21]. In addition, *I. farinosa* could also lead the ghost moth larvae to death by being surface-treated with conidia suspension of *I. farinosa* (Figure S6).

## 5. Conclusions

The composition of the ghost moth gut microbiota is unique when compared with other insects. The results demonstrated that gut microbiota of the wild and laboratory-reared ghost moth populations greatly differed. The wild ghost moth larvae had a significantly higher abundance of *Carnobacterium* bacteria and *Ophiocordyceps* fungi in the gut compared with the laboratory-reared larvae. The gut of the laboratory-reared larvae was enriched by *Wolbachia* and other opportunistic pathogenic bacteria such as *Serratia*, *Pseudomonas,* and *Flavobacterium,* as well as opportunistic pathogenic fungi *Aspergillus* and *Candida*. These findings will help improve the effectiveness of the laboratory-reared *Thitarodes* hosts for the cultivation of the Chinese cordyceps, by selecting beneficial probiotics from the gut microbiota.

## Figures and Tables

**Table 1:** Group information of the 16S rRNA and ITS gene analyses.

Group Name ^1^	Altitude (Masl)	Environmental Temperature ^2^	Major Diet ^3^	Sampling Location
W.SD (W)	~4128	Wild, −20~10 °C	plant roots of alpine meadow	Shade Town, Kangding City, Ganzi Tibetan Autonomous Prefecture, Sichuan Province, China
W.GG (W)	~3958	Wild, −20~10 °C	plant roots of alpine meadow	Hailuogou, Gongga mountain, Moxi Town, Luding County, Ganzi Tibetan Autonomous Prefecture, Sichuan Province, China
W.XJ (W)	~3823	Wild, −20~10 °C	plant roots of alpine meadow	Xiaojin County, Aba Tibetan and Qiang Autonomous Prefecture, Sichuan Province, China
A.SD (A)	~43	Artificial rearing at 8 °C	roots of *P. anserina*	Haizhu District, Guangzhou City, Guangdong Province, China
A.GG (A)	~43	Artificial rearing at 8 °C	roots of *P. anserina*	Haizhu District, Guangzhou City, Guangdong Province, China
A.XJ (A)	~43	Artificial rearing at 8 °C	roots of *P. anserina*	Haizhu District, Guangzhou City, Guangdong Province, China

^1^ A.SD, A.GG and A.XJ larvae were artificially reared in the laboratory from the pupae of W.SD, W.GG, and W.XJ, respectively; ^2^ The environment temperature is cited from the reference [46]; ^3^ More than 100 species in 19 families of host plants for the wild ghost moth larvae [47].

**Table 2:** Bacterial and fungal species in the gut of the wild and laboratory-reared *Thitarodes* populations by culture-dependent method.

Category	Microbe Species	Phylum	Population Sampled					
			W.SD	W.GG	W.XJ	A.SD	A.GG	A.XJ
Bacteria	*Acinetobacter lwoffii*	Proteobacteria	+					+
	*Aeromonas* sp.	Proteobacteria		+	++			
	*Agromyces* sp.	Actinobacteria	+					
	*Arthrobacter* sp.	Actinobacteria	+			+	+	+
	*Bacillus mycoides*	Firmicutes			+			
	*Bacillus* sp.	Firmicutes		+		+		+
	*Buttiauxella* sp.	Proteobacteria			+			
	*Carnobacterium maltaromaticum*	Firmicutes	++++	++++	++++	+++	+++	+++
	*Chryseobacterium* sp.	Bacteroidetes	+		+	+	+	+
	*Enterococcus* sp.	Firmicutes		++				+
	*Glutamicibacter* sp.	Actinobacteria	+			+		
	*Microbacterium foliorum*	Actinobacteria		+				
	*Microbacterium* sp.	Actinobacteria	+		+	+	+	++
	*Oerskovia* sp.	Actinobacteria		+				
	*Pantoea* sp.	Proteobacteria		+				
	*Pseudomonas* sp.	Proteobacteria	+	+	+	++	+	+
	*Pseudomonas fragi*	Proteobacteria	+		+	+		+
	*Pseudoclavibacter* sp.	Actinobacteria	+					
	*Rahnella aquatilis*	Proteobacteria	+	+	+	+	+	+
	*Raoultella terrigena*	Proteobacteria	+	+	+			
	*Rhodococcus* sp.	Actinobacteria	+		+			+
	*Serratia fonticola*	Proteobacteria		+	+			++
	*Serratia plymuthica*	Proteobacteria	+	+		+	+	
	*Serratia proteamaculans*	Proteobacteria		+			+	
	*Staphylococcus* sp.	Firmicutes			+	+	+	+
	*Streptomyces* sp.	Actinobacteria	+	+	+	+	+	+
Fungi	*Apiotrichum* sp.	Basidiomycota		+				
	*Aspergillus* sp.	Ascomycota	+		+			+
	*Candida* sp.	Ascomycota	++	++		+	+	
	*Chaetomium* sp.	Ascomycota		+	+			
	*Cladosporium* sp.	Ascomycota	+	+	+	+		
	*Mucor hiemalis*	Mucoromycota		+				
	*Mucor racemosus*	Mucoromycota	+				+	
	Nectriaceae	Ascomycota		+	+			+
	*Penicillium polonicum*	Ascomycota			+			
	*Penicillium* sp.	Ascomycota	+		+			+
	*Rhodotorula* sp.	Basidiomycota		+				
	*Trichoderma* sp.	Ascomycota		+				
	Sporocadaceae	Ascomycota			+			
	*Sterigmatomyces halophilus*	Basidiomycota		+		+		
	**Bacterial species**		**15**	**14**	**14**	**12**	**10**	**14**
	**Fungal species**		**5**	**9**	**7**	**3**	**2**	**3**

W.SD, W.GG, and W.XJ larval populations were collected from the high-altitude alpine meadow in Sichuan, China. A.SD, A.GG, and A.XJ larvae were artificially reared in the laboratory from the pupae of W.SD, W.GG, and W.XJ populations, respectively. +, ≤100 colonies detected in the plates; ++, > 100 ≤ 1000 colonies detected in the plates; +++, > 1000 ≤ 2500 colonies detected in the plates; ++++, >2500 colonies detected in the plates.

## Data Availability

All the data analyzed has been submitted to the database with ac-cession number described in this article.

## Supplementary Materials

The following are available online at https://www.mdpi.com/article/10.3390/insects12040327/s1: Figure S1: The geographical locations of the ghost moths sampled from the Tibetan alpine meadow in Google Earth 7.1; Figure S2: The phylogenetic tree of the ghost moth constructed by the mitochondrial cytochrome b gene using Neighbor-joining methods; Figure S3: Numbers of effective sequences and OTUs in each sample for bacteria (A) and fungi (B); Figure S4: Rank-abundance and rarefaction curves for all samples; Figure S5: PCR amplification on genomic DNA of the six groups with specific primers of *Isaria farinose*; Figure S6: Infection characteristics of the ghost moth caused by surface-treated with 10^8^ spores per ml of *I. farinosa*; Table S1: The percentages of the microbe species isolated from five different media at 13 °C and 23 °C; Table S2: Sequencing summary for bacteria and fungi; Table S3: Comparison of the indices of alpha diversity between groups using Turkey’s test; Table S4: Dissimilarity comparison of microbial community structures between group; Table S5: ITS analyses of isolates from *Thitarodes* sp. cadavers during the artificial cultivation.
