# Supplementary material for: Gut Bacterial and Fungal Communities of the Wild and Laboratory-Reared Thitarodes Larvae, Host of the Chinese Medicinal Fungus Ophiocordyceps sinensis on Tibetan Plateau
Source: Insects. 2021 Apr 7;12(4):327. doi: 10.3390/insects12040327 (PMC8067570; doi:10.3390/insects12040327)
Supplement: Supplementary file 1 [file insects-12-00327-s001.pdf]

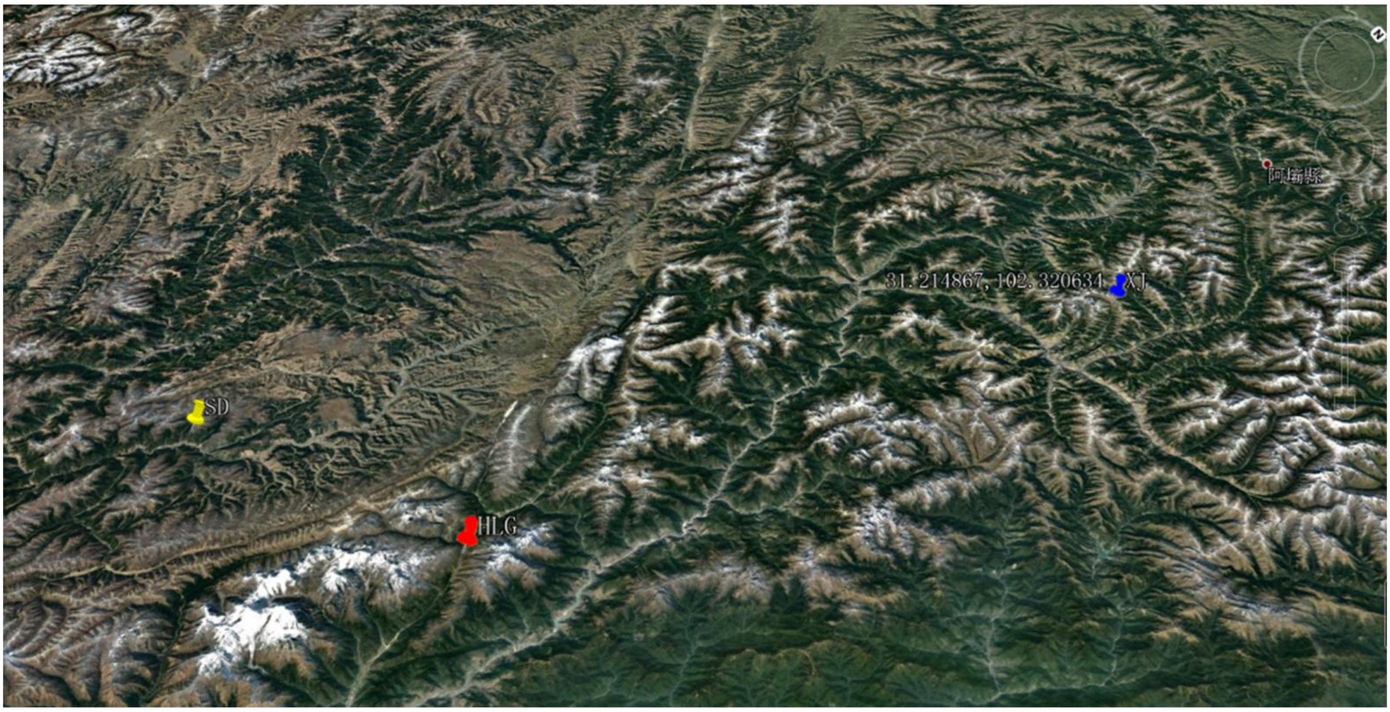

Figure S1. The geographical locations for the sampling ghost moth populations from the Tibetan alpine meadow in Google Earth 7.1. SD population (yellow color arrow): collected from Shade Town (101°22´E, 29°40´N, 4128.61 meters), Kangding City, Ganzi Tibetan Autonomous Prefecture, Sichuan Province; GG population (red color arrow): collected from Hailuogou, Gongga moutain (102°0´E, 29°54´N, 3957.92 meters), Moxi Town, Luding County, Ganzi Tibetan Autonomous Prefecture, Sichuan Province; XJ population (blue color arrow): collected from Xiaojin County (102°19´E, 31°12´N, 3823.33 meters), Aba Tibetan and Qiang Autonomous Prefecture, Sichuan Province.

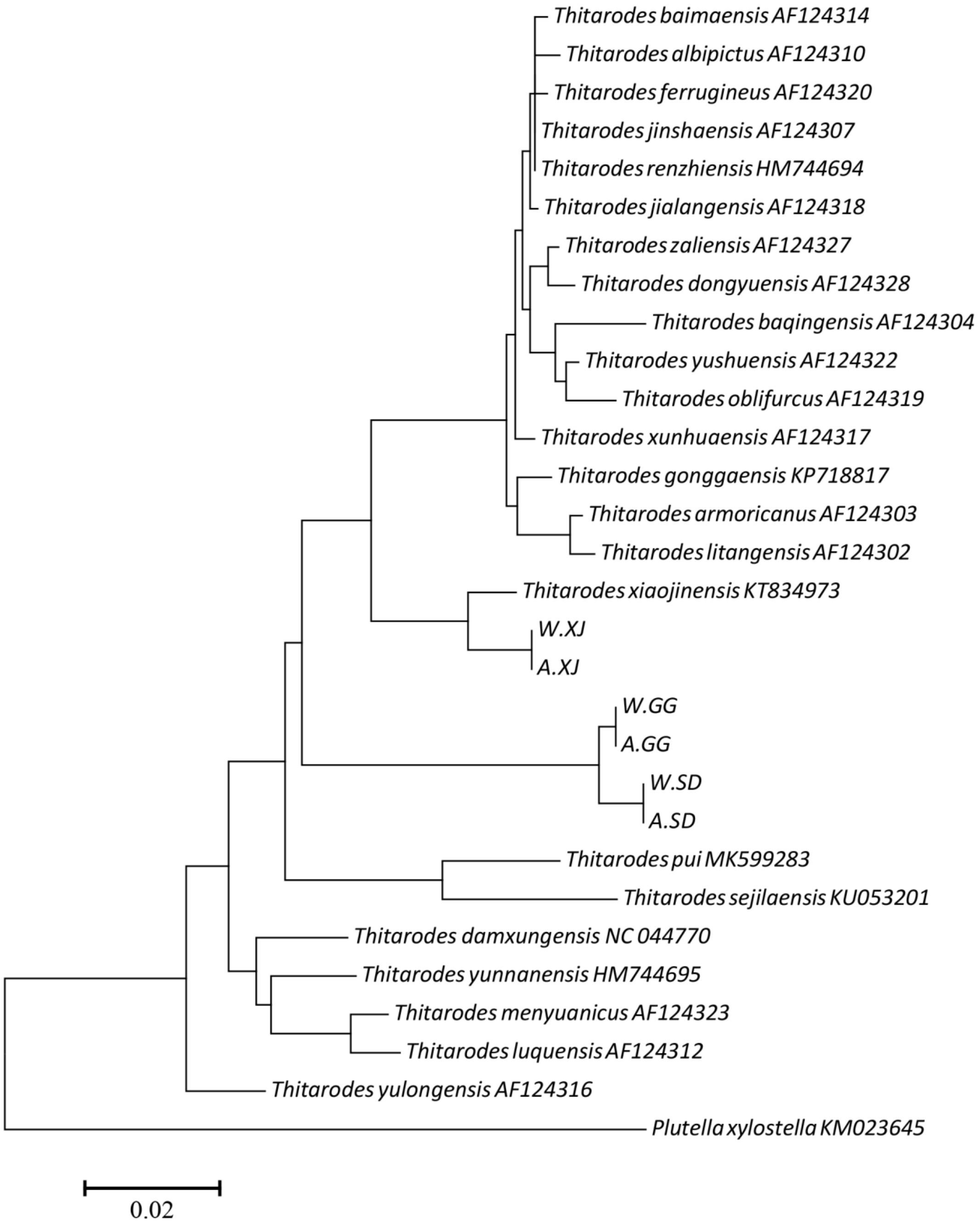

Figure S2. The phylogenetic tree of the ghost moth constructed by the mitochondrial cytochrome b gene using Neighbor-joining methods. Populations W.SD, W.GG and W.XJ larvae were collected from the high-altitude alpine meadow in Sichuan, China. Populations A.SD, A.GG and A.XJ larvae were reared in the low-altitude laboratory in Guangzhou, China from sterilized embryos of W.SD, W.GG and W.XJ correspondingly.

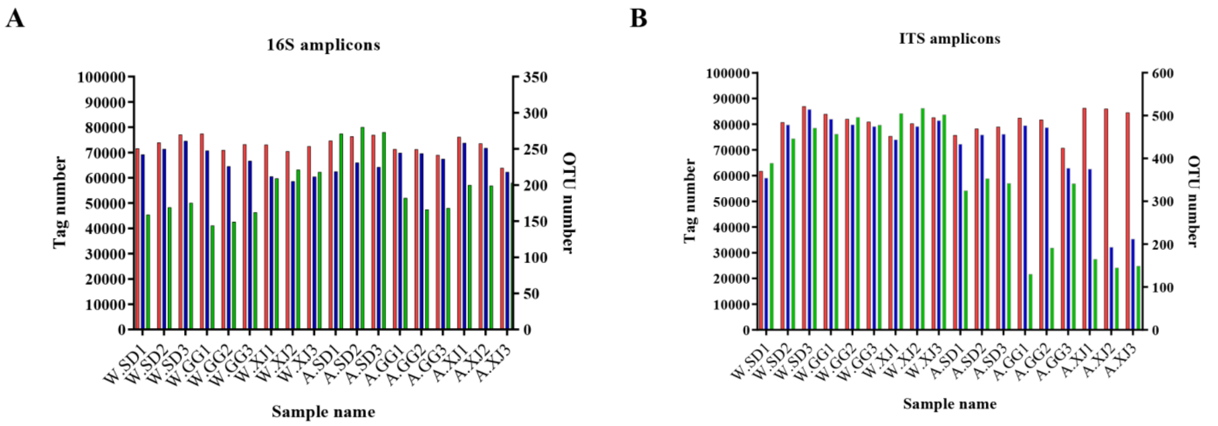

Figure S3. Numbers of effective sequences and OTUs in each sample for bacteria (**A**) and fungi (**B**). Effective tags (red) were sequences without low-quality sequences and chimeras, and were used for annotation and other analyses. Taxon tags (blue) represent the sequences that could be clustered into OTUs and annotated.

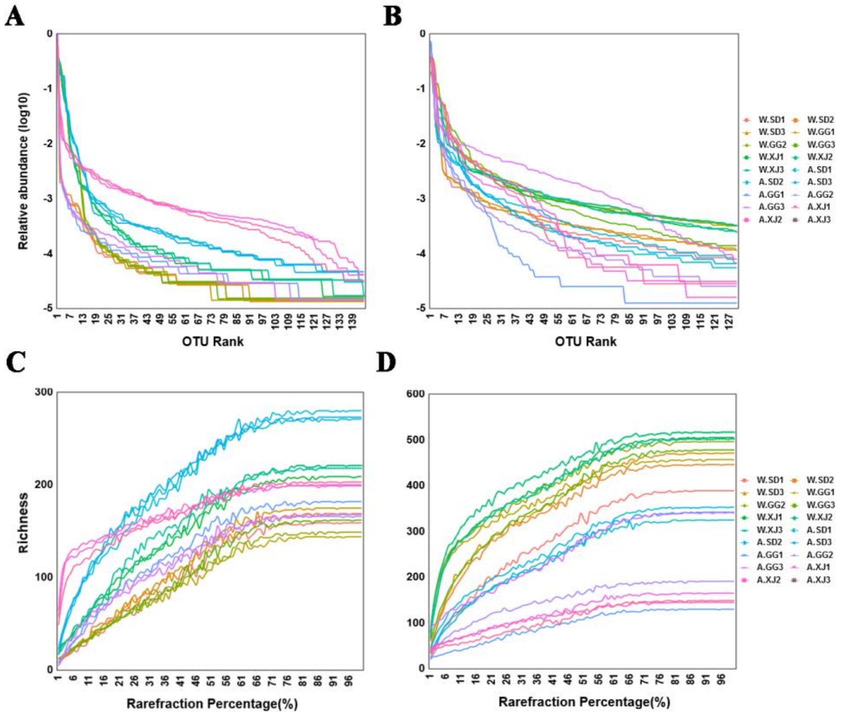

Figure S4. Rank-abundance and rarefaction curves for all samples. Rank-abundance curves for bacteria (**A**) and fungi (**B**); Rarefaction curves for bacteria (**C**) and fungi (**D**).

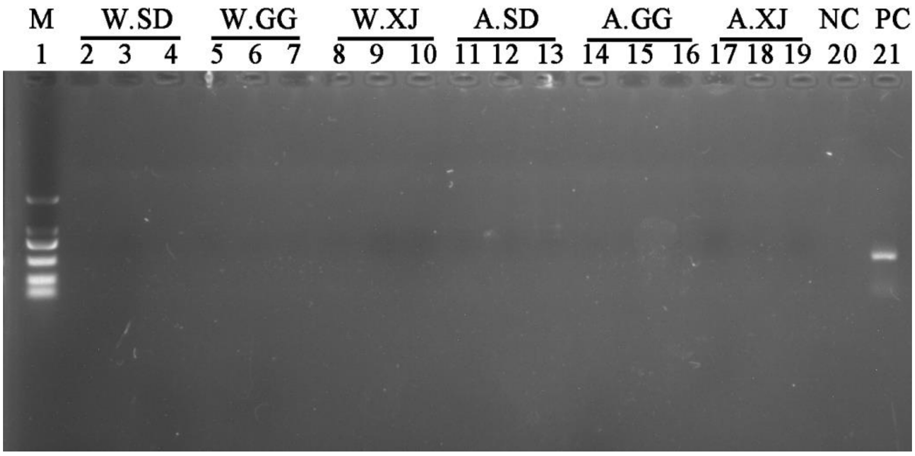

Figure S5. PCR amplification on genomic DNA of the six ghost moth populations with specific primers of *Isaria farinosa*.

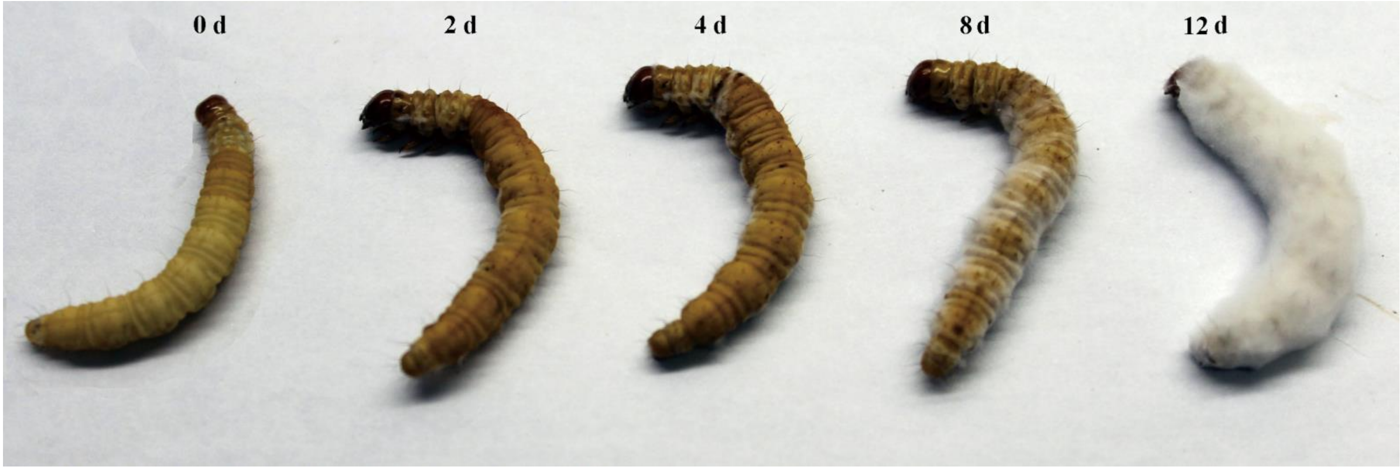

Figure S6. Infection characteristics of the ghost moth caused by surface-treated with 10^8 spores per ml of *Isaria farinosa*.

Table S1. The percentages of the microbe species isolated from five different media at 13°C and 23 °C.

**Media**	**Percentage of the isolated microbe species from different samples** [1]					
	**W.SD** [2]	**W.GG**	**W.XJ**	**A.SD**	**A.GG**	**A.XJ**
LB	45.00	45.45	47.37	46.67	41.67	41.18
TSB	55.00	54.55	57.89	53.33	58.33	58.82
HIA	45.00	45.45	47.37	46.67	41.67	47.06
GSA	40.00	40.91	42.11	40.00	41.67	41.18
PPDA	55.00	63.64	63.16	60.00	58.33	58.82
Temperature (°C)						
13	65.00	59.09	47.37	60.00	58.33	52.94
23	90.00	86.36	89.47	93.33	91.67	88.23

[1] Percentage of the isolated microbe species from each medium or each temperature in each sample =Numbers of bacterial and fungal species isolated from each medium or each temperature in each sample / total numbers of bacterial and fungal species isolated from all five media or two temperatures in each sample. [2] W.SD2, W.GG, W.XJ, A.SD, A.GG and A.XJ indicated ghost moths from six different populations.

Table S2. Sequencing summary for bacteria and fungi.

**Taxonomy**	**Sequencing**	**Sample ID**	**Raw total reads**	**Clean total reads**	**Clean total tags**	**Q20(%)**	**Q30(%)**	**GC(%)**
Bacteria	paired-end	W.SD1	86000	85698	71577	99.3	96.8	53.3
	paired-end	W.SD2	89000	88707	73884	99.3	96.8	53.3
	paired-end	W.SD3	92000	91731	77062	99.4	96.9	53.3
	paired-end	W.GG1	92000	91678	77409	99.4	97	53.6
	paired-end	W.GG2	85000	84704	70991	99.4	96.9	53.6
	paired-end	W.GG3	87000	86733	73234	99.4	97	53.6
	paired-end	W.XJ1	86000	85748	73057	99.4	97.1	53.5
	paired-end	W.XJ2	84000	83711	70482	99.4	97	53.5
	paired-end	W.XJ3	86000	85704	72469	99.4	97	53.5
	paired-end	A.SD1	89000	88710	74717	99.4	97	51.6
	paired-end	A.SD2	91000	90728	76379	99.4	97	51.6
	paired-end	A.SD3	93000	92660	76926	99.4	96.9	51.6
	paired-end	A.GG1	88000	87686	71318	99.3	96.5	48.1
	paired-end	A.GG2	89000	88683	71217	99.3	96.4	48.1
	paired-end	A.GG3	85000	84720	69002	99.3	96.5	48.2
	paired-end	A.XJ1	91000	90704	76159	99.4	97	53.4
	paired-end	A.XJ2	87000	86724	73567	99.4	97	53.3
	paired-end	A.XJ3	76529	76217	63903	99.4	97	53.4
		**Total**	**1576529**	**1571246**	**1313353**			
Fungi	paired-end	W.SD1	90000	79268	61743	99.9	99.4	47.8
	paired-end	W.SD2	86000	85797	80742	99.9	99.5	46.7

paired-end	W.SD3	92000	91863	86930	99.9	99.5	47
paired-end	W.GG1	93000	92456	83932	99.9	99.3	46.2
paired-end	W.GG2	89000	88785	81993	99.9	99.3	46.1
paired-end	W.GG3	87000	86839	80936	99.9	99.2	47.2
paired-end	W.XJ1	84000	83740	75316	99.9	99.3	46.1
paired-end	W.XJ2	89000	88777	80257	99.9	99.3	46.1
paired-end	W.XJ3	92000	91764	82580	99.9	99.3	46.4
paired-end	A.SD1	90000	87525	75711	99.9	99.6	39.7
paired-end	A.SD2	85000	84839	78229	99.9	99.6	40
paired-end	A.SD3	86000	85831	78973	99.9	99.6	39.6
paired-end	A.GG1	89615	89008	82406	99.8	98.7	36
paired-end	A.GG2	86000	85871	81766	99.8	99	37
paired-end	A.GG3	84000	83732	70721	99.9	99.3	43.1
paired-end	A.XJ1	91000	90874	86237	99.8	99.1	46.6
paired-end	A.XJ2	92000	91874	86017	99.7	98.4	53.8
paired-end	A.XJ3	90322	90146	84486	99.7	98.5	52.3
	**Total**	**1595937**	**1578989**	**1438975**			

Table S3. Comparison of the indices of alpha diversity between ghost moth populations using Turkey's test.

Sequencing	Paired comparison	Richness		Chao1		Simpson	
		p	Significance	*p*	Significance	*p*	Significance
16S	A.GG-A.SD	0.000	***	0.000	***	0.000	***
	A.GG-A.XJ	0.024	*	0.027	*	0.000	***
	A.GG-W.GG	0.051		0.078		0.000	***
	A.GG-W.SD	0.562		0.659		0.042	*
	A.GG-W.XJ	0.003	**	0.004	**	0.000	***
	A.SD-A.XJ	0.000	***	0.001	**	0.000	***
	A.SD-W.GG	0.000	***	0.000	***	0.000	***
	A.SD-W.SD	0.000	***	0.000	***	0.000	***
	A.SD-W.XJ	0.000	***	0.000	***	0.000	***
	A.XJ-W.GG	0.009	**	0.012	*	0.000	***
	A.XJ-W.SD	0.015	*	0.017	*	0.000	***
	A.XJ-W.XJ	0.040	*	0.027	*	0.000	***
	W.GG-W.SD	0.089		0.116		0.000	***
	W.GG-W.XJ	0.001	**	0.002	**	0.000	***
	W.SD-W.XJ	0.002	**	0.002	**	0.000	***
ITS	A.GG-A.SD	0.196		0.199		0.382	
	A.GG-A.XJ	0.393		0.398		0.262	
	A.GG-W.GG	0.051		0.053		0.147	
	A.GG-W.SD	0.061		0.064		0.547	
	A.GG-W.XJ	0.044	*	0.046		0.211	
	A.SD-A.XJ	0.000	***	0.000	***	0.002	**
	A.SD-W.GG	0.001	**	0.001	**	0.000	***
	A.SD-W.SD	0.047	*	0.049	*	0.001	**
	A.SD-W.XJ	0.000	***	0.001	**	0.000	***
	A.XJ-W.GG	0.000	***	0.000	***	0.030	*
	A.XJ-W.SD	0.005	**	0.005	**	0.042	*
	A.XJ-W.XJ	0.000	***	0.000	***	0.249	
	W.GG-W.SD	0.223		0.225		0.007	**
	W.GG-W.XJ	0.095		0.102		0.074	
	W.SD-W.XJ	0.091		0.095		0.000	***

Notes: The asterisks indicated significant difference at $p < 0.05$ (*), $p < 0.01$ (**) and $p < 0.001$ (***).

Table S4. Dissimilarity comparison of microbial community structures among six ghost moth populations.

Sequencing	Paired comparison	ANOSIM		Adonis	
		R-value	*p*-value	R^2	Pr(>F)
16S	A.GG vs A.SD	1	0.1	0.999	0.1
	A.GG vs A.XJ	1	0.1	0.989	0.1
	A.GG vs W.GG	1	0.1	0.999	0.1
	A.GG vs W.SD	1	0.1	0.999	0.1
	A.GG vs W.XJ	1	0.1	0.999	0.1
	A.SD vs A.XJ	1	0.1	0.982	0.1
	A.SD vs W.GG	1	0.1	0.997	0.1
	A.SD vs W.SD	1	0.1	0.999	0.1
	A.SD vs W.XJ	1	0.1	0.998	0.1
	A.XJ vs W.GG	1	0.1	0.984	0.1
	A.XJ vs W.SD	1	0.1	0.99	0.1
	A.XJ vs W.XJ	1	0.1	0.973	0.1
	W.GG vs W.SD	1	0.1	0.998	0.1
	W.GG vs W.XJ	1	0.1	0.995	0.1
	W.SD vs W.XJ	1	0.1	0.998	0.1
	A vs W	0.611	0.001	0.309	0.001
ITS	A.GG vs A.SD	0.667	0.1	0.553	0.1
	A.GG vs A.XJ	0.63	0.1	0.499	0.1
	A.GG vs W.GG	0.593	0.1	0.4	0.2
	A.GG vs W.SD	0.889	0.1	0.643	0.1
	A.GG vs W.XJ	0.778	0.1	0.646	0.1
	A.SD vs A.XJ	0.889	0.1	0.81	0.1
	A.SD vs W.GG	0.556	0.1	0.585	0.1
	A.SD vs W.SD	1	0.1	0.971	0.1
	A.SD vs W.XJ	1	0.1	0.99	0.1
	A.XJ vs W.GG	0.704	0.1	0.535	0.1
	A.XJ vs W.SD	1	0.1	0.841	0.1
	A.XJ vs W.XJ	1	0.1	0.845	0.1
	W.GG vs W.SD	0.556	0.1	0.605	0.1
	W.GG vs W.XJ	0.556	0.1	0.579	0.1
	W.SD vs W.XJ	1	0.1	0.947	0.1
	A vs W	0.45	0.001	0.252	0.001

Table S5. ITS analyses of fungal isolates from the mummificated *Thitarodes* sp. during the artificial cultivation.

Fungal species	Number (total 300)	Proportion (%)
Isaria farinosa (*Paecilomyces farinosus*)	244	81.33
Penicillium polonicum	17	5.67
Penicillium commune	11	3.67
Beauveria bassiana	8	2.67
Mucor hiemalis	8	2.67
Mucor racemosu	7	2.33
Fusarium sp.	5	1.67
